# Clinical Features of Early Stage COVID-19 in a Primary Care Setting

**DOI:** 10.3389/fmed.2021.764884

**Published:** 2021-11-23

**Authors:** Yohei Kawatani, Kei Nakayama, Atsushi Sawamura, Koichi Fujikawa, Motoki Nagai, Takaki Hori

**Affiliations:** ^1^Department of Cardiovascular Surgery, Kamagaya General Hospital, Kamagaya, Japan; ^2^Department of Emergency Medicine, Kamagaya General Hospital, Kamagaya, Japan; ^3^Department of Gastrointestinal Surgery, Kamagaya General Hospital, Kamagaya, Japan

**Keywords:** COVID-19, primary care, predictive value, predictive score, SARS-CoV-2, monocyte-to-neutrophile ratio

## Abstract

**Background:** The coronavirus disease 2019 (COVID-19) pandemic remains a global healthcare crisis. Nevertheless, the majority of COVID-19 cases involve mild to moderate symptoms in the early stages. The lack of information relating to these cases necessitates further investigation.

**Methods:** Patients visiting the outpatient clinic at the Kamagaya General Hospital were screened by interview and body temperature check. After initial screening, severe acute respiratory syndrome coronavirus 2 (SARS-CoV-2) infection was suspected in 481 patients who then underwent blood tests and the loop-mediated isothermal amplification (LAMP) test for SARS-CoV-2. Clinical characteristics between positive and negative SARS-CoV-2 groups were compared. Further, the novel predictive value of routine blood test results for SARS-CoV-2 infection was evaluated using ROC analysis.

**Results:** A total of 15,560 patients visited our hospital during the study period. After exclusion and initial screening by interview, 481 patients underwent the LAMP test and routine blood tests. Of these patients, 69 (14.3%) were positive for SARS-CoV-2 and diagnosed with COVID-19 (positive group), and 412 (85.7%) were negative (negative group). The median period between the first onset of symptoms and visit to our hospital was 3.4 and 2.9 days in the negative and positive groups, respectively. Cough (*p* = 0.014), rhinorrhea (*p* = 0.039), and taste disorders (*p* < 0.001) were significantly more common in the positive group, while gastrointestinal symptoms in the negative group (*p* = 0.043). The white blood cell count (*p* < 0.001), neutrophil count (*p* < 0.001), and percentage of neutrophils (*p* < 0.001) were higher in the negative group. The percentage of monocytes (*p* < 0.001) and the levels of ferritin (*p* < 0.001) were higher in the positive group. As per the predictive values for COVID-19 using blood tests, the values for the area under the curve for the neutrophil-to-monocyte ratio (NMR), white blood cell-to-hemoglobin ratio (WHR), and the product of the two (NMWH) were 0.857, 0.837, and 0.887, respectively.

**Conclusion:** Symptoms in early stage COVID-19 patients were similar to those in previous reports. Some blood test results were not consistent with previous reports. NMR, WHR, and NMWH are novel diagnostic scores in early-stage mild-symptom COVID-19 patients in primary care settings.

## Introduction

In December 2019, the novel coronavirus disease 2019 (COVID-19), caused by severe acute respiratory syndrome coronavirus 2 (SARS-CoV-2), was reported in Wuhan, China (1). The rapid spread of COVID-19 to other countries created a major public health crisis (2) and led the World Health Organization (WHO) to declare COVID-19 a global pandemic in May 2020 (3).

Despite the presence of extensive data concerning COVID-19 in hospitalized patients and those admitted to emergency departments (4–6), there is a paucity of information focusing on COVID-19 patients with mild symptoms in outpatient clinics. The majority of infected individuals present with mild to moderate symptoms, which hampers the early detection of the disease (7, 8) and drives the transmission of infection (9). In primary care settings, many detection tests cannot be performed, and the results cannot be obtained immediately (10–12). In such situations, it is useful to identify patients highly suspected of having COVID-19 from those who visit primary care clinics. Therefore, we aimed to report on the clinical features of early stage COVID-19 and to establish simple diagnostic values using routine blood tests to improve point-of-care diagnosis and treatment of COVID-19 in a primary care setting.

## Materials and Methods

### Patient Inclusion Criteria and Diagnosis of COVID-19

We performed this retrospective study to evaluate the clinical features of patients with COVID-19 who visited the Kamagaya General Hospital between January 1 and 31, 2021. All patients were interviewed (questioner list is shown in 2.1.1), and if they had any signs suggesting COVID-19 (fever above 37.5°C, cough, myalgia, headache, fatigue, rhinorrhea, sputum, taste disorder, gastrointestinal symptoms, and a history of exposure to COVID-19 patients) (13, 14), they underwent routine blood tests (Table 1) and a SARS-CoV-2 loop-mediated isothermal amplification (LAMP) test (Loopamp^®^, Eiken Chemical Co., Ltd. Tokyo). A positive COVID-19 diagnosis was made based on LAMP test results. Clinical features of the SARS-CoV-2-positive and -negative patient groups were compared. In addition, we attempted to establish simple predictive values for patients with mild COVID-19 symptoms using routine blood test results.

**Table 1:** Patient characteristics, vital signs, and symptoms upon presentation to the outpatient clinic.

	**SARS-CoV-2 negative group (*****n*** **=** **412)**		**SARS-CoV-2 positive group (*****n*** **=** **69)**		***p*** **-value**
	**Mean ± SD**	**Median (25th−75th percentile)**	**Mean ± SD**	**Median (25th−75th percentile)**	
Age	47.31 ± 30.15	44 (30.25–64)	46.55 ± 16.97	44 (33–61)	0.955
Days of visit after the onset	3.42 ± 4.65	3 (1–4)	2.95 ± 2.20	3 (1–5)	0.209
Highest body temperature before the visit	37.54 ± 0.94	37.5 (37–38.075)	37.78 ± 0.88	37.9 (37.1–38.4)	0.045
Body temperature at the visit	36.78 ± 0.65	36.7 (36.4–37)	36.81 ± 0.66	36.7 (36.4–37.2)	0.657
Systolic blood pressure	127.29 ± 20.18	127 (112–139)	135.53 ± 19.22	134 (118–147)	0.002
Diastolic blood pressure	81.89 ± 14.30	81 (72–90)	89.09 ± 13.52	89 (81–99)	<0.001
Heart rate	86.43 ± 15.71	86 (74–96)	85.78 ± 14.12	83 (77–92)	0.845
SpO_2_	97.31 ± 1.87	98 (97–98)	97.29 ± 1.21	98 (97–98)	0.221
**Symptoms**	**Total number**	**SARS-CoV-2 negative group (** ***n*** **, %)**	**SARS-CoV-2 positive group (** ***n*** **, %)**	***p*** **value**	
Fever	362	308 (74.8)	54 (78.3)	0.651	
Cough	192	156 (37.9)	36 (52.2)	0.014	
Myalgia	3	2 (0.5)	1 (1.4)	0.372	
Arthralgia	110	89 (21.6)	21 (30.4)	0.121	
Headache	170	147 (35.7)	23 (33.3)	0.786	
Fatigue	176	152 (36.9)	24 (34.8)	0.788	
Rhinorrhea	162	131 (31.8)	31 (44.9)	0.039	
sore throat	174	148 (35.9)	26 (37.7)	0.788	
Sputum	15	11 (2.7)	4 (5.8)	0.249	
Taste disorder	31	16 (3.9)	15 (21.7)	<0.001	
Dyspnea	52	46 (11.2)	6 (8.7)	0.677	
Gastrointestinal symptoms	112	103 (25.0)	9 (13.0)	0.043	
Exposure history	42	24 (5.8)	18 (26.1)	<0.001	

*SpO_2_, peripheral capillary oxygen saturation*.

#### Screening Questions

The screening questions were:

- What are your symptoms?
- Do you have any of the following symptoms: cough, aching muscles, headache, tiredness, runny nose, sputum, taste disorder, digestive problems?
- When did your first symptom start?
- Have you had contact with anyone with COVID-19 in the past 2 weeks?
- Do you know your highest temperature before this visit? (If yes:) What was it??

### Statistical Analysis

All statistical analyses were performed using IBM SPSS for Macintosh, version 28 (IBM Corp., Armonk, NY, USA). Quantitative variables were expressed as mean ± standard deviation and median (interquartile range 2575%). Qualitative variables were expressed as frequencies and percentages in each group. Symptoms and blood test results of SARS-CoV-2-positive and -negative patients were analyzed for differences using the Mann-Whitney test or chi-square test. Receiver operating characteristic curve (ROC) analysis was performed to compare predictive values and optimal cut-off values to determine SARS-CoV-2 positivity. The level of statistical significance for all analyses was set at *p* < 0.05.

### Ethical Approval and Patient Consent

This study was approved by the institutional review board (Approved number: TGE01745-64). We applied an opt-out method to obtain patient consent for participation and publication according to the guideline set by the institutional review board.

## Results

**Figure 1:**
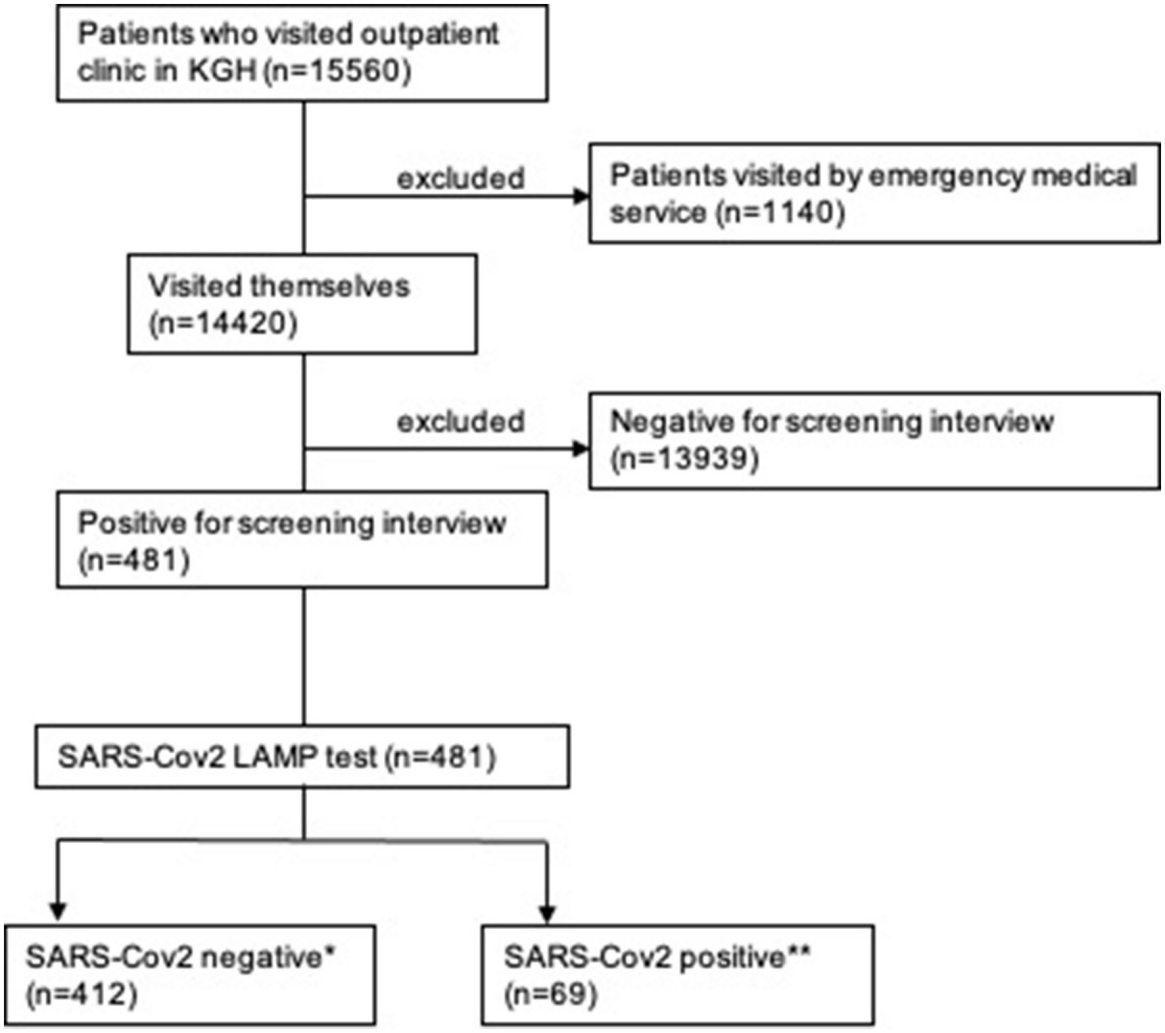
Flow diagram of the inclusion and exclusion criteria used in this study. *negative group, **positive group. KGH: Kamagaya General Hospital.

A total of 15,560 patients visited our hospital during the study period. After excluding 1,140 patients who arrived for emergency medical treatment, the remaining 14,420 patients who visited our outpatient clinic were screened by interview. Based on initial screening, 481 patients underwent the LAMP test and diagnostic blood tests. Subsequently, 69 (14.3%) patients tested positive for SARS-CoV-2 and were diagnosed with COVID-19 (positive group), while 412 (85.7%) were negative for SARS-CoV-2 (negative group) (Figure 1).

The mean and median values for the number of days from the first onset of symptoms to presentation to our outpatient clinic were 3.4 ± 4.6 and 3 (1–4) days in the negative group, and 2.9 ± 2.2 and 3 (1–5) days in the positive group, respectively. The mean and median ages were 47.2 ± 20.2 and 44 (30–64) years in the negative group and 46.5 ± 17.0 and 44 (33–61) years in the positive group, respectively. There were no significant differences between the two groups. Exposure history was observed more frequently in the positive group (18, 26.1%) than in the negative group (24, 5.8%). Body temperature, heart rate, and oxygen saturation (SpO_2_) were not significant. Blood pressure tended to be higher in the positive group than in the negative group. Cough, rhinorrhea, and taste disorders were observed more frequently in the positive group, but gastrointestinal symptoms were prevalent in the negative group (Table 1).

Routine blood test results and biochemical parameters were assessed (Table 2). White blood cell count and platelet count were higher in the negative group, while red blood cell count and hemoglobin levels were higher in the positive group. Neutrophil, eosinophil, and basophil counts and percentages were lower in the positive group. A low lymphocyte count but high lymphocyte percentage was noted in the positive group. Transaminase, gamma-glutamyl transferase, and estimated glomerular filtration rate was lower in the positive group than in the negative group. Conversely, creatinine, uric acid, and ferritin levels were higher in the positive group. Chloride and calcium ion levels were lower in the positive group, but sodium and potassium ion levels were not significantly different between the two groups. Total bilirubin, C-reactive protein, and procalcitonin levels were significantly higher in the negative group. All blood test results in the positive group were within normal ranges.

**Table 2:** Patient blood test results, biochemical parameters, and predictive scores.

	**SARS-CoV-2 negative group**		**SARS-CoV-2 positive group**		***p*** **-value**
	**Mean ± SD**	**Median (25–75th percentile)**	**Mean ± SD**	**Median (25–75th percentile)**	
**Complete blood cell counts**					
White blood cells/μL	78.88 ± 35.26	71 (54.25–92.75)	51.36 ± 14.81	50 (41–57)	<0.001
Red blood cells/μL	479.19 ± 62.50	481 (443–522.75)	507.09 ± 45.63	505 (477–537)	<0.001
Hemoglobin (mg/dl)	14.32 ± 1.80	14.4 (13.1–15.7)	15.35 ± 1.18	15.2 (14.6–16.4)	<0.001
Platelets (× 10^∧^4/μL)	26.62 ± 7.29	26 (22.025–30.6)	21.24 ± 5.46	20.5 (16.4–24.8)	<0.001
Monocyte (%)	5.79 ± 1.87	5.55 (4.5–6.8)	9.73 ± 3.45	8.9 (7–11.7)	<0.001
Monocyte count/μL	4,412.12 ± 2,171.59	3,750 (2,908–5,445)	4,963.27 ± 2,271.57	4,459 (3,312–5,985)	0.329
Neutrophil (%)	68.81 ± 11.89	69.5 (60.575–77.775)	60.10 ± 11.99	60.6 (49.8–70.5)	<0.001
Neutrophil count/μL	56,834.43 ± 33,374.47	4,459 (3,312–5,985)	31,804.75 ± 13,551.80	28,952 (19,890–40,392)	<0.001
Lymphocyte (%)	22.74 ± 10.43	22.25 (14.6–30.2)	28.12 ± 11.46	28.3 (20.0–34.9)	<0.001
Lymphocyte count/μL	15,790.27 ± 6,212.05	15,369.5 (11,855–19,763)	13,498.13 ± 4,597.18	14,025 (10,438–15,756)	0.003
Basophil (%)	0.53 ± 0.33	0.5 (0.3–0.7)	0.39 ± 0.24	0.4 (0.2–0.6)	<0.001
Basophil count/μL	377.30 ± 217.86	327 (216–491)	198.89 ± 128.45	188 (102–300)	<0.001
Eosinophil (%)	2.14 ± 2.33	1.4 (0.6–2.8)	1.66 ± 1.62	1.1 (0.5–2.5)	0.078
Eosinophil count/μL	1,469.21 ± 1,857.46	973 (473–1,782)	898.60 ± 978.24	520 (204–1,113)	<0.001
**Chemicals**					
CRP (mg/dL)	2.56 ± 4.29	0.6 (0.05–3.3275)	0.81 ± 1.30	0.33 (0.18–0.85)	0.373
CK (U/L)	100.69 ± 62.50	86 (58.25–119)	110.82 ± 60.67	91 (65–157)	0.349
AST (U/L)	26.76 ± 66.75	20 (16–25)	28.33 ± 9.89	27 (20–35)	<0.001
ALT (U/L)	26.98 ± 33.17	19 (13–29)	32.62 ± 25.53	27 (18–35)	<0.001
LDH (U/L)	185.11 ± 74.12	173.5 (152–201)	192.40 ± 39.21	185 (170–216)	0.027
ALP (U/L)	234.28 ± 132.61	213 (172–264.75)	225.00 ± 60.71	212 (182–252)	0.635
GGT (U/L)	43.00 ± 85.05	24 (16.25–38)	49.22 ± 38.94	37 (22–63)	<0.001
AMY (U/L)	78.86 ± 45.96	72 (56–90)	67.44 ± 24.60	64 (50–77)	0.092
TP (g/dL)	7.46 ± 0.53	7.5 (7.1–7.8)	7.53 ± 0.47	7.6 (7.2–7.8)	0.84
ALB (g/dL)	4.37 ± 0.48	4.5 (4.2–4.7)	4.50 ± 0.36	4.5 (4.3–4.7)	0.189
A/G ratio	1.45 ± 0.28	1.445 (1.26–1.67)	1.52 ± 0.28	1.48 (1.38–1.7)	0.104
T-Bil (mg/dL)	0.82 ± 0.41	0.7 (0.6–1)	0.69 ± 0.27	0.7 (0.5–0.8)	0.007
BUN (mg/dL)	13.76 ± 7.32	12.3 (9.925–15.5)	13.55 ± 3.98	13 (10.7–15.4)	0.451
Crea (mg/dL)	0.89 ± 0.82	0.77 (0.65–0.91)	0.89 ± 0.27	0.9 (0.74–0.97)	0.001
eGFR (mL/min/1.73 m^2^)	76.36 ± 19.61	77.8 (65.3–89.675)	72.30 ± 17.35	70 (64–77)	0.007
UA (mg/dL)	5.03 ± 1.45	4.8 (4–5.9)	5.65 ± 1.54	5.5 (4.5–6.6)	0.013
Na (mmol/L)	140.52 ± 2.63	141 (139–142)	140.82 ± 2.07	141 (140–142)	0.984
K (mmol/L)	4.18 ± 0.43	4.2 (3.9–4.4)	4.24 ± 0.40	4.2 (4–4.5)	0.932
Cl (mmol/L)	102.50 ± 2.89	103 (101–104)	101.75 ± 2.23	102 (100–103)	0.032
Ca (mmol/L)	9.40 ± 0.43	9.4 (9.1–9.7)	9.37 ± 0.38	9.4 (9.2–9.6)	0.032
Glu (mg/dL)	109.91 ± 34.35	101 (90–115)	113.47 ± 43.49	99 (93–116)	0.306
FER (ng/mL)	152.87 ± 173.79	108 (53–204)	245.85 ± 190.86	178 (132–331)	<0.001
PCT (ng/mL)	0.21 ± 1.07	0.02 (0.01–0.06)	0.03 ± 0.02	0.03 (0.02–0.04)	0.725
**Predictive scores**					
NLR	5.06 ± 6.55	1.20 (1.45–3.18)	2.80 ± 2.31	1.45 (1.05–2.17)	<0.001
PLR	209.52 ± 270.92	130.38 (107.84–170.00)	178.75 ± 95.44	119.94 (99.25–164.98)	0.528
LMR	4.15 ± 2.32	2.36 (1.52–3.81)	3.40 ± 1.94	2.11 (1.53–2.86)	0.002
NMR	14.54 ± 24.38	9.48 (7.35–12.29)	7.15 ± 2.86	5.15 (3.30–6.76)	<0.001
WHR	5.74 ± 2.83	3.78 (3.05–5.00)	3.26 ± 0.92	2.62 (2.19–3.07)	<0.001
NMWH	94.47 ± 209.20	39.28 (24.78–58.90)	24.10 ± 13.21	14.28 (8.90–21.40)	<0.001

*CRP, c-reactive protein; CK, creatine kinase; AST, alanine aminotransferase; AST, aspartate aminotransferase; LDH, lactate dehydrogenase; ALP, alkaline phosphatase; GGT, gamma-glutamyl transferase; AMY, amylase; TP, total protein; ALB, albumin; A/G ratio, albumin-globulin ratio; T-Bil, total- bilirubin; BUN, blood urea nitrogen; Crea, creatinine; eGFR, estimated glomerular filtration rate; UA, uric acid; Na, Sodium; K, potassium; Cl, chloride; Ca, calcium; Glu, glucose; FER, ferritin; PCT, procalcitonin; NLR, neutrophil to lymphocyte ratio; PLR, platelet-to-lymphocyte ratio; LMR, lymphocyte-to-monocyte ratio; NMR, neutrophile-to-monocyte ratio; WHR, white blood cell-to-hemoglobin ratio; NMWH, product of NMR and WHR*.

**Figure 2:**
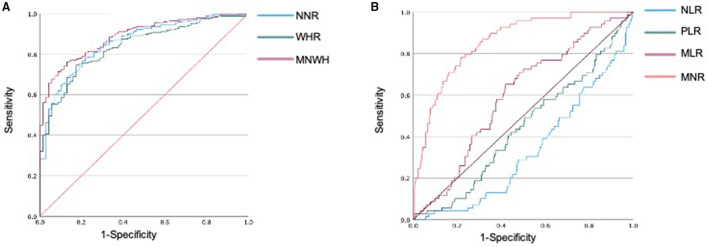
Receiver operating characteristic (ROC) analysis to evaluate the diagnostic value of hematologic markers for COVID-19. **(A)** Diagnostic values of neutrophils and lymphocytes in differentiating SARS-CoV-2 positive patients from negative patients. **(B)** Diagnostic values of NLR, MLR, and PLR in differentiating SARS-CoV-2-positive patients from SARS-CoV-2-negative patients. NLR, neutrophil-to-lymphocyte ratio; PLR, platelet-to-lymphocyte ratio; LMR, lymphocyte-to-monocyte ratio; NMR, neutrophil-to-monocyte ratio; WHR, white blood cell-to-hemoglobin ratio; NMWH, product of NMR and WHR.

The area under the ROC curve (AUC) of neutrophil-to-monocyte ratio was 0.857 (95% CI, 0.814–0.900, *p* < 0.001). At a neutrophil-to-monocyte ratio cut-off point of ≤7.45, sensitivity and specificity were 90.0 and 56.5%, respectively. At an neutrophil-to-monocyte ratio cut-off point of ≤8.87, sensitivity and specificity were 80.1 and 73.9%, respectively. The AUC of white blood cell-to-hemoglobin ratio was 0.837 (95% CI, 0.793–0.881, *p* < 0.001). At a white blood cell-to-hemoglobin ratio cut-off point of ≤3.06, sensitivity and specificity were 90.3 and 52.2%, respectively. At a white blood cell-to-hemoglobin ratio cut-off point of ≤35.7, sensitivity and specificity were 80.1 and 78.3%, respectively (Figure 2A). The AUC of the product of neutrophil-to-monocyte ratio and white blood cell-to-hemoglobin ratio (NMWH) was 0.887 (95% CI, 0.853–0.921, *p* < 0.001). At a NMWH cut-off point of ≤25.0, sensitivity and specificity were 90.0 and 67.8%, respectively. At a NMWH cut-off point of ≤35.7, sensitivity and specificity were 80.1 and 78.3%, respectively (Table 3). The neutrophil-to-lymphocyte ratio, platelet-to-lymphocyte ratio, and lymphocyte-to-monocyte ratio were not effective diagnostic values in this study (Figure 2B).

**Table 3:** Receiver operating characteristic (ROC) curve analysis of predictive scores and cut-off points.

**Test result variables**	**Area under the curve**	**Std. error**	***p*** **-value**	**95% confidence interval**	**Cut off point**	**Sensitivity (%)**	**Specificity (%)**
NMR	0.857	0.022	0.000	0.814–0.90	7.45	90.0	56.5
					8.87	80.1	73.9
WHR	0.837	0.022	0.000	0.793–0.881	3.06	90.3	52.2
					3.57	80.3	69.9
NMWH	0.887	0.017	0.000	0.853–0.921	25	90.0	67.8
					35.7	80.1	78.3
NLR	0.667	0.033	0.000	0.602–0.732			
PLR	0.558	0.036	0.125	0.487–0.628			
LMR	0.596	0.034	0.010	0.529–0.663			

*NMR, neutrophile-to-monocyte ratio; WHR, white blood cell-to-hemoglobin ratio; NMWH, product of NMR and WHR; NLR, neutrophil to lymphocyte ratio; PLR, platelet-to-lymphocyte ratio; LMR, lymphocyte-to-monocyte ratio*.

## Discussion

### Diagnosis Technique

Currently, reverse transcription polymerase chain reaction (RT-PCR) is the gold standard for diagnosing COVID-19 (15, 16). However, it is expensive and time consuming (11). LAMP is a rapid, sensitive, cost-effective method (17, 18). In a meta-analysis by Anita et al., the LAMP test demonstrated a cumulative sensitivity of 95.5% (CI 97.5%, 90.8–97.9%) and cumulative specificity of 99.5% (CI 97.5%, 97.7–99.9%) for RT-PCR results (19). In our hospital, the LAMP test is used for diagnosing SARS-CoV-2 in the outpatient clinic. Though RT-PCR and LAMP are appropriate methods for detecting SARS-CoV-2 in the clinical setting, RT-PCR reportedly has a sensitivity of 70–90% (12, 20, 21). The possible reasons for the low efficiency of viral nucleic acid detection may include (1) subpar development of nucleic acid detection technology, (2) variations in detection rates of different manufacturers, (3) low viral load, or (4) improper specimen sampling. Further, Fang et al. reported that the sensitivity of RT-PCR for detecting SARS-CoV-2 was 71% (21). These findings support the idea of reduced reliance on RT-PCR or LAMP tests in the early stages of COVID-19 and show that there is potential for a better diagnostic method using blood tests.

Precise evaluation of the possibility of COVID-19 positivity among patients suggested of having early stage COVID-19 can improve primary care. We assume that it is highly useful if the evaluation is conducted through interviews and/or routine blood tests. This method can improve determining pre-test probability and enables us to identify patients strongly suggested for undergoing the RT-PCR or LAMP test.

### Patient Background and Present Histories

Almost all patients with COVID-19 visited our hospital 3 days after the first onset of symptoms. In this study, the clinical features of the SARS-CoV-2-positive group represented mild symptoms and early stage COVID-19. We compared SARS-CoV-2-positive and -negative groups in order to identify useful features for predicting COVID-19 among suspected COVID-19 patients in primary care settings.

There are reports stating that SARS-CoV-2 positivity is associated with older age (22, 23), and other studies report that age is not associated with the disease (24). In this study, age was not significantly different between the SARS-CoV-2-positive and -negative groups. One of the reasons for the difference in results among reports was assumed to be the difference between the patients examined in this study and previous studies. This study concludes that in early stage COVID-19 patients with mild symptoms, age was not a factor.

SARS-CoV-2 may be transmitted via aerosols or fomites (1). The most common forms of transmission are droplets and physical contact (25). Therefore, exposure history is a vital factor. In our study, exposure history was higher in the SARS-CoV-2-positive group (18, 26.1% of patients). In a study conducted in China in February 2020, at a time when COVID-19 was endemic to the region, 27.8% of patients exhibited a history of exposure (26). Thus, exposure history is important for the detection of COVID-19 with mild symptoms.

### Symptoms

Symptoms of the SARS-CoV-2 infection reportedly resemble those of the SARS-CoV infection (27, 28). While the mortality of COVID-19 is 1/10th that of SARS, SARS-CoV-2 has a much greater transmissibility rate; thus, it may be a greater threat to global health (29). The respiratory tract and lungs are the main targets of SARS-CoV-2 (30), and the symptoms of infection include mainly fever, cough, and fatigue/myalgia (31). In our study, about half of the positive group presented with cough (36, 52.2%, *p* = 0.014) and rhinorrhea (31, 44.9%, *p* = 0.039), which was significantly higher than that of the negative group. On the other hand, sputum production was not significantly different between both groups. It was revealed that early stage COVID-19 patients presented mainly with upper respiratory tract symptoms.

In previous reports, gastrointestinal (GI) symptoms were rarer than in other viral infections (4, 28, 31). A systematic review reported that the prevalence of diarrhea, nausea/vomiting, and abdominal pain in patients with COVID-19 were 9.1, 5.2, and 3.5%, respectively (32). In our study, all GI symptoms were observed in 13 and 25% of the positive and negative groups, respectively; this difference was significant (*p* = 0.043). We assume that the prevalence of symptoms in the early stages of COVID-19 is the same as noted in previous reports. Symptoms were observed more frequently in the negative group, possibly because the negative group included patients with diseases that mainly affected the GI system, such as cholecystitis or infective enteritis.

Further investigation after the pandemic revealed taste disorders in patients with COVID-19. Taste disorders can be an initial symptom or early manifestation of the disease (33, 34).

In an epidemiological survey of taste and olfactory disorders in COVID-19 patients, taste disorder was observed in 56.2% of the PCR-positive group, which was significantly higher than 13.9% of the negative group (35). In our study, the prevalence rate was lower than in other reports in both the positive (15, 21.7%) and negative groups (16, 3.9%, *p* < 0.001). However, the result that taste disorder was significantly more common in the positive group was consistent. We can conclude that taste disorder manifesting in early stage COVID-19 may have diagnostic utility. In general, the symptoms in the present study were consistent with those in the previous studies, although their magnitudes were different.

### Blood Tests

Anemia has been reported to have developed with increasing severity of COVID-19 (36). Moreover, anemia may be associated with worse mortality rates (37). In the current study, hemoglobin [15.2 (14.6–16.4)] was within normal range in the positive group and lower in the negative group. Either the positive or the negative group has a tendency for anemia.

White blood cell count and differentiation were within the normal range in the positive group. Neutrophil count and percentage were lower in the positive group. Monocyte count did not show a significant difference, but the percentage was higher in the positive group. Lymphocyte count was lower, but lymphocyte percentage was higher in the positive group. The negative group had a tendency for leukocytosis, with increased neutrophils, whereas the positive group had almost normal test results. These results were not consistent with the previous reports. In many reports, lymphocytopenia has been observed in COVID-19 patients (38, 39). The lymphocyte count in this study did not show any significant abnormality. Other studies reported that there was no significant change in monocyte count in COVID-19 patients (40). This reported result matches with our study results. Eosinophil and basophil levels have reportedly been low in COVID-19 patients (41). Our study also showed the same result. However, the mechanism and magnitude of these parameters remain unclear. These differences between previous reports and the reports of this study may be because of the severity in patients. We focused on the patients with mild stages of COVID-19 in our study.

The levels of CRP (C-reactive protein) did not differ between the two groups. In the positive group, the CRP level was 0.33 (0.18–0.85), which was within the normal range. High CRP levels have reportedly been associated with severity and progression to pneumonia in COVID-19 patients (42, 43). In our study, the subjects with mild COVID-19 demonstrated normal CRP levels. In contrast to CRP levels, the ferritin levels were significantly higher in the positive than in the negative group (44). Ferritin has been reported to reflect systemic inflammation (45, 46). We assume that SARS-CoV-2 may cause inflammation in different ways than other infectious or inflammatory diseases in the negative group.

Transaminase and bilirubin levels were higher in the positive group than in the negative group, although the results were almost within the normal range. The elevation of liver enzymes in COVID-19 patients has been previously reported (47). In addition, coexisting liver failure is associated with increased mortality (48), and liver injury is associated with worse COVID-19 prognosis (32). Qi et al. reported that liver cholangiocytes were angiotensin-converting enzyme II (ACE2)-enriched cells (49); however, pathological findings indicated that SARS-CoV-2 was not observed in the liver of patients with COVID-19 (30). These reports indicate that the mechanism and importance of liver injury in COVID-19 patients is unclear.

Kidney injury has been reported in patients with COVID-19 and is associated with increased severity of symptoms and mortality (50). Chen et al. reported that ACE2, which is a receptor of SARS-CoV-2, is mainly expressed by different cell types in the human kidney. This indicates that SARS-CoV-2 mediates acute kidney injury (51). There are a few reports on renal function in the early stages of COVID-19. This study shows that the renal function tends to be lower in the positive group, although the magnitude is small. Thus, kidney injury may occur even in the early stages of COVID-19.

There are reports on the abnormality of ion levels in COVID-19 patients, but the significance of the abnormality is unclear. Dubey et al. reported that calcium levels were lower in patients with mild symptoms but higher in patients with moderate symptoms. They did not detect abnormalities in the levels of sodium and potassium ions (52). Our study shows that the chloride (*p* = 0.032) and calcium (*p* = 0.032) ion levels are slightly lower in the positive group than in the negative group. However, the implications of these findings are yet to be determined.

### Prognostic Values

We attempted to explore new diagnostic values for mild COVID-19. In our study, neutrophil-to-monocyte ratio, white blood cell-to-hemoglobin ratio, and their product showed high AUC in the ROC analysis. Herein, neutrophil-to-monocyte ratio represents a lower neutrophil count and a higher monocyte percentage in the positive group than in the negative group. The white blood cell-to-hemoglobin ratio represents higher hemoglobin and lower white blood cell counts in the positive group than in the negative group. Moreover, NMWH may provide a more accurate score. There are a few reports regarding these scores pertaining to COVID-19 diagnosis. There are several other diagnostic scores for COVID-19, such as neutrophile-to-lymphocyte ratio, lymphocyte-to-monocyte ratio, and platelet-to-lymphocyte ratio (22, 24, 26, 53–56). Prior to the COVID-19 pandemic, these scores were used in the evaluation of malignant tumors (57, 58). However, the scores were not significantly useful in our current study. One of the reasons may be the change in the differentiation of white blood cells in the course of COVID-19 (39). The neutrophil-to-monocyte ratio, white blood cell-to-hemoglobin ratio, and NMWH novel diagnostic scores are used for screening early-stage mildly symptomatic COVID-19 patients who visited the outpatient clinic, as well as for suspected COVID-19 cases. He et al. proposed diagnostic values for the SARS-CoV-2 PCR test using routine blood tests and machine learning (23). Their model showed an AUC of 0.854. The optimal cut-off value with the maximum Youden's index showed sensitivity and specificity of 0.761 and 0.808, respectively. Interestingly, our simple formula using routine blood tests also had a high AUC as theirs.

We assume that our prognostic scores can identify upper respiratory tract viral infections, which includes COVID-19, in the patients suspected of having COVID-19 after screening in the outpatient clinic. Peng et al. reported that the predictive values could distinguish COVID-19 patients from healthy participants; however, they could not distinguish COVID-19 patients from the patients with pneumonia caused by influenza (24). The same may be the case with our study.

### Study Limitations

There were some limitations to the current study. The sensitivity of the method used in the study of early stage COVID-19 can vary between 70 and 90%. Further, this study was conducted retrospectively in a single facility. Additionally, almost all patients in the study were Japanese. Further larger studies in multiple different populations are required in order to check the generalizability of these results. Despite these limitations, we believe that our diagnostic values have the potential to increase pre-test probability or identify highly suspected COVID-19 patients in outpatient clinics, improving primary care medicine.

## Data Availability Statement

The original contributions presented in the study are included in the article/supplementary material, further inquiries can be directed to the corresponding author/s.

## Ethics Statement

The studies involving human participants were reviewed and approved by the Tokushukai Group Ethics Committee. Written informed consent for participation was not required for this study in accordance with the national legislation and the institutional requirements.

## Author Contributions

YK and TH were involved in the study conception and design. YK conducted all statistical analyses and wrote the manuscript. All authors were involved in patient treatment and contributed and approved the final manuscript.

## Conflict of Interest

The authors declare that the research was conducted in the absence of any commercial or financial relationships that could be construed as a potential conflict of interest.

## Publisher's Note

All claims expressed in this article are solely those of the authors and do not necessarily represent those of their affiliated organizations, or those of the publisher, the editors and the reviewers. Any product that may be evaluated in this article, or claim that may be made by its manufacturer, is not guaranteed or endorsed by the publisher.
